# A Pre-Grasping Motion Planning Method Based on Improved Artificial Potential Field for Continuum Robots

**DOI:** 10.3390/s23229105

**Published:** 2023-11-10

**Authors:** Lihua Wang, Zezhou Sun, Yaobing Wang, Jie Wang, Zhijun Zhao, Chengxu Yang, Chuliang Yan

**Affiliations:** 1 School of Mechanical and Aerospace Engineering, Jilin University, Changchun 130025, China; wlh20@mails.jlu.edu.cn (L.W.);; 2 Beijing Institute of Spacecraft System Engineering, Beijing 100094, China

**Keywords:** active debris removal, continuum robots, whole-arm grasping, pre-grasping motion planning, artificial potential field

## Abstract

Secure and reliable active debris removal methods are crucial for maintaining the stability of the space environment. Continuum robots, with their hyper-redundant degrees of freedom, offer the ability to capture targets of varying sizes and shapes through whole-arm grasping, making them well-suited for active debris removal missions. This paper proposes a pre-grasping motion planning method for continuum robots based on an improved artificial potential field to restrict the movement area of the grasping target and prevent its escape during the pre-grasping phase. The analysis of the grasping workspace ensures that the target is within the workspace when starting the pre-grasping motion planning by dividing the continuum robot into delivery and grasping segments. An improved artificial potential field is proposed to guide the continuum robot in surrounding the target and creating a grasping area. Specifically, the improved artificial potential field consists of a spatial rotating potential field, an attractive potential field incorporating position and posture potential fields, and a repulsive potential field. The simulation results demonstrate the effectiveness of the proposed method. A comparison of motion planning results between methods that disregard and consider the posture potential field shows that the inclusion of the posture potential field improves the performance of pre-grasping motion planning for spatial targets, achieving a success rate of up to 97.8%.

## 1. Introduction

Due to the increasing amount of space debris (SD) resulting from space launch activities, orbital resources are becoming limited, and the risk of collisions is rising, posing a significant safety threat to operational spacecraft. The Inter-Agency Space Debris Coordination Committee (IADC) has emphasized the need for immediate action to prevent the proliferation of SD [1]. Active Debris Removal (ADR) methods offer an effective solution to remove large space debris [2].

ADR methods can be categorized into capture removal methods and non-capture removal methods. Capture removal techniques that have been proposed include robotic arms [3,4,5,6,7,8,9], net capturing [10,11,12,13], harpoon [10,14,15,16,17], tethered systems [18,19,20], and flexible capturing [21,22,23,24]. Non-capture removal techniques include laser propulsion removal, ion beam removal, and other similar technologies to reduce the orbit height of SD [25,26,27,28,29]. Among these techniques, using robotic arms with suitably configured end effectors is more mature [30]. However, SD is a non-cooperative target with diverse characteristics. The limited degrees of freedom make it challenging for rigid robotic arms to satisfy the requirements of target adaptability, motion flexibility, and task safety for grasping operations. To overcome these limitations, researchers have explored the utilization of continuum robots (CRs) for grasping SD.

CRs are biomimetic robots inspired by octopus tentacles and elephant trunks [31]. Their flexibility, adaptability, and safety make them promising for a wide range of applications such as medical surgery [32,33], inspection and repair for aerospace [34], and flexible grasping [35,36]. In addition to grasping with end-effectors, their flexible backbone enables them to wrap around targets, which is known as whole-arm grasping. Current research on whole-arm grasping by CRs primarily focuses on bionic structures. Numerous studies have developed various CR prototypes, and the capability of whole-arm grasping has been verified [37,38,39]. However, few studies focus on CR motion planning for whole-arm grasping. Jinglin Li proposed algorithms for autonomous whole-arm grasping operations, which include determining grasping configurations and progressively grasping to generate force-closure grasps in open and cluttered environments [40,41,42]. Camilla Agabiti introduced a whole-arm grasping strategy inspired by the elephant trunk, utilizing the contact points identified on the object to control the deformation of the soft arm [43]. However, the studies mentioned above do not consider the influence of the environment in the grasping process. On Earth, successful grasping can be achieved without considering pre-grasping due to the presence of friction. However, the process of capturing SD occurs in a microgravity and vacuum environment, where even a small force can cause significant motion of the target [44]. Ignoring the effects of the space environment may lead to premature contact, resulting in the target drifting away from the CR and potentially creating more SD. Therefore, it is necessary to consider the motion planning for CRs in conjunction with the space environment for ADR missions.

Current research on motion planning for CRs primarily relies on inverse kinematics solutions. Various methods have been proposed, such as Jacobian pseudo-inverse methods [45,46,47], geometrical methods [48,49,50], and learning-based algorithms [51]. Offline planning algorithms, like RRT and A*, have also been utilized for CR path-planning tasks [52,53,54]. However, ADR missions are characterized by high levels of uncertainty and multiple time-varying factors. Coupled with the limited on-satellite computing resources, the aforementioned methods pose challenges, as they necessitate complex computing and regenerated planning when applied to different targets or dynamic environments. This can potentially hinder the effectiveness of the mission execution [55]. The artificial potential field (APF), an online path planning method [56], presents a promising alternative due to its computational efficiency, wide applicability, and ability to generate smooth paths, making it particularly valuable for ADR missions. However, when applied to CRs, the complexity of the potential field and motion planning increases due to the multiple degrees of freedom. Ahmad Atakaiu improved the traditional APF by integrating a novel attractive potential field in the actuator space to avoid the limits of the mechanical design [57]. Nevertheless, this may lead the robot to be trapped in the local solution. In response to this issue, Yu Tian developed a virtual guiding pipeline (VGP) within a guided potential field for continuum manipulators, which solves the local minimum problem and allows navigation through narrow gaps [58]. Linjia Hao tackled the local minimum and unreachable issues with an improved APF, including the piecewise repulsion function and dynamic gravitational constant [59]. Yinchu Wang prevented the robot from getting trapped in the local extrema and deviating from the desired path by introducing a rotating potential field near obstacles [60]. For whole-arm grasping using CRs with force closure grasps, a specific grasping point is unnecessary, as the CR merely needs to conform to the target’s shape to achieve force closure. This advantage is due to the hyper-redundancy of the CR. Conventional planning methods for CRs typically rely on knowledge of the target’s grasping configuration, which underutilizes the adaptability of the CR. An automatic and universal grasping motion planning method could better leverage the advantages of CRs.

In this paper, we present a pre-grasping motion planning method for the CR based on an IAPF that considers the characteristics of the space environment. In the pre-grasping phase, the CR is directed by potential fields to encircle the grasping target and confine its movement to a certain area without predetermined grasping configurations, which lays the foundation for subsequent safe and reliable grasping operations. Our method consists of several steps. First, we analyze the grasping workspace of the CR to ensure that the target is within the grasping workspace at the beginning. Once the pre-grasping phase is initiated, an IAPF is constructed with the target information and robot configuration. The proposed IAPF consists of three potential fields: a spatial rotating potential field used to guide the end of the CR to encircle the target, an attractive potential field to accelerate the process and improve the performance of pre-grasping motion planning, and a repulsive potential field established around the target to prevent contact between the CR and the target until the grasping area is achieved. Then, simulations are conducted to demonstrate the effectiveness of the proposed method. The main contributions are summarized as follows:

1. A pre-grasping motion planning method based on an IAPF is proposed for CR to encircle the grasping target in space. As usual, both goal configurations and motion planning are required for whole-arm grasping. In this paper, a rotating potential field is integrated so that motion planning is implemented without predetermined goal configurations.
2. A posture attractive potential field based on the end position of a segment of a CR is proposed, and it improves the performance of pre-grasping motion planning for spatial grasping targets in terms of success rate and execution times.

The remainder of this paper is organized as follows. Section 2 provides a brief introduction to the structure and the kinematics of the CR. Section 3 presents the pre-grasping motion planning method based on an IAPF for the CR, following an introduction to the grasping workspace and the grasping area involved. Section 4 presents the results of the numerical simulations conducted to demonstrate the effectiveness of the proposed pre-grasping motion planning method. Section 5 concludes the paper.

## 2. Mechanism Design and Kinematic Modeling of the CR

### 2.1. Mechanism Design

**Figure 1:**
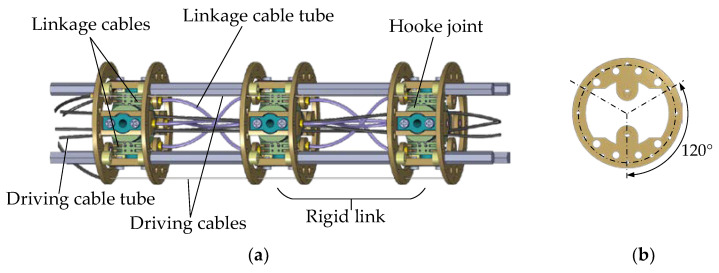
Mechanical design of CR. (**a**) Diagram of one segment; (**b**) the distribution of cable holes.

In this paper, a linkage cable-driven CR with rigid links is designed for ADR. The main structure of the CR consists of four identical segments connected in series, and the structure of each segment is illustrated in Figure 1. Each segment is composed of rigid links, Hooke joints, driving cables, and linkage cables. The Hooke joint allows adjacent rigid links to rotate about two axes. Each driving cable is wrapped with a driving cable tube, with one end fixed to the cable hole and the other end attached to the driving control box. The motion of the driving cables of each segment is decoupled by the tubes wrapping around the driving cables. Additionally, the synchronization of the joint motion within each segment is achieved by the linkage cables, which ensure that adjacent joints move at the same angle. The parameters of the designed CR are listed in Table 1.

### 2.2. Kinematic Modeling

**Figure 2:**
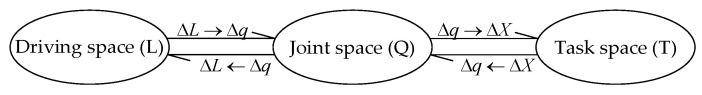
Multilevel kinematic mappings of the CR.

The CR is an indirectly driven robot with multilevel mapping relationships. Figure 2 shows the multilevel kinematic mappings of the CR, which relate the length of the driving cables to the end pose (position and posture) of the CR through joint angles.

#### 2.2.1. Mapping between Joint Space and Task Space

**Figure 3:**
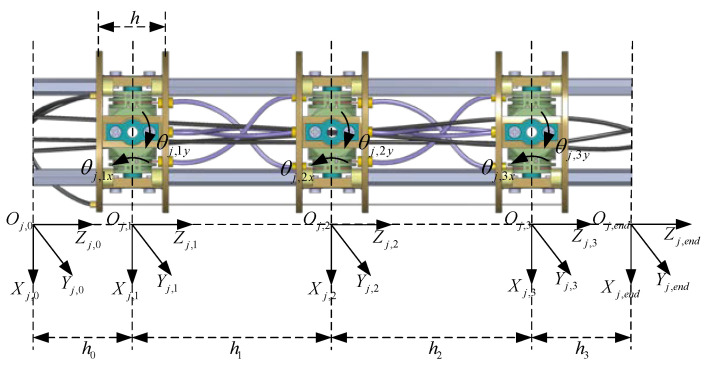
The coordinate systems of segment j.

To establish the relationship between the joint angles and the end pose of the CR, a coordinate system is defined for each link. The coordinate systems of segment j are shown in Figure 3, while the coordinate systems of the other segments are identical to segment j. The base frame, {j,0}(j=1,2,3,4), is fixed at the center of the base link; the frame {j,i}(i=1,2,3) is established at the center of joint i; and the end frame, {j,end}, is defined at the center of the link at the end of segment j. It can be obtained that the frames {j+1,0} and {j,end} are equal while being located relative to the frame {1,0}. The joint i connects the adjacent links i−1 and i. The length of each link is denoted as $h_{i}$, and the initial distance between the two cable holes of adjacent links is h. The link i rotates around the two rotation axes $X_{j,i}$ and $Y_{j,i}$, and the joint angles are denoted as $\theta_{j,ix}$ and $\theta_{j,iy}$, respectively. Due to the utilization of linkage cables, the joint angles within segment j can always remain equal, where $\theta_{j,ix}=q_{j,x}$ and $\theta_{j,iy}=q_{j,y}$.

According to the definitions above, the homogeneous transformation matrix from {j,i} to {j,i−1} is

(1) $${}^{j,i-1}T_{j,i}=Trans_{z}(h_{i-1})\cdot Rot_{x}(q_{j,x})\cdot Rot_{y}(q_{j,y})(i=1,2,3)$$

Then, the homogeneous transformation matrix from the base frame to the end frame of segment j can be obtained as

(2) $${}^{j,0}T_{j,end}{=}^{j,0}T_{j,1}\cdot^{j,1}T_{j,2}\cdot^{j,2}T_{j,3}\cdot^{j,3}T_{j,end}$$

where ${}^{j,3}T_{j,end}=Trans_{z}(h_{3})$. Then, the end frame with respect to the base frame of the CR can be expressed as

(3) $${}^{0}T_{end}{=}^{0}T_{1,end}\cdot^{1,end}T_{2,end}\cdot^{2,end}T_{3,end}\cdot^{3,end}T_{4,end}$$

#### 2.2.2. Mapping between Driving Space and Joint Space

**Figure 4:**
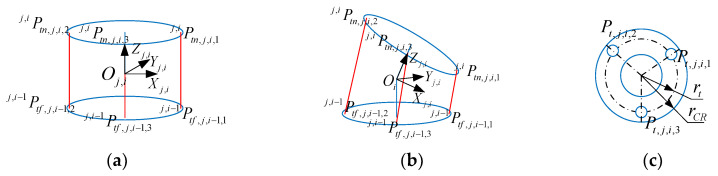
Kinematic model of a single joint. (**a**) Initial state; (**b**) final state; (**c**) the distribution of the cable holes.

Figure 4 illustrates the kinematic model of a single joint, where the two rotation angles of joint i in segment j are driven by a driving cable, $C_{j,k}\left(k=1,2,3\right)$. The relationship between the joint angles and the length of the driving cable can be calculated through the position of the cable holes. The cable holes are distributed evenly along a circular path with a radius of $r_{t}$, spaced at intervals of 120°. Specifically, the cable hole $P_{tf,j,i-1,k}\left(k=1,2,3\right)$ is located on the disk of link i−1, which is further away from the CR’s end, while the cable hole $P_{tn,j,i,k}$ is located on the disk of link i, which is closer to the CR’s end. Additionally, $P_{tf,j,i-1,k}$ is opposite to $P_{tn,j,i,k}$ of joint i.

The position of the cable holes of link i in segment j can be expressed as

(4) $${}^{j,i}P_{tn,j,i,k}={\left[r_{t}\cdot \cos(\frac{\pi }{6}+\frac{2\pi }{3}(k-1)),r_{t}\cdot \sin(\frac{\pi }{6}+\frac{2\pi }{3}(k-1)),\frac{h}{2}\right]}^{T}(k=1,2,3)$$

(5) $${}^{j,i}P_{tf,j,i,k}={\left[r_{t}\cdot \cos(\frac{\pi }{6}+\frac{2\pi }{3}(k-1)),r_{t}\cdot \sin(\frac{\pi }{6}+\frac{2\pi }{3}(k-1)),h_{i}-\frac{h}{2}\right]}^{T}(k=1,2,3)$$

Then, the length of the driving cable, $C_{j,k},$ between the two disks of joint i in segment j can be obtained:

(6) $$l_{j,i,k}=\left\|{}^{j,i-1}P_{tn,j,i,k}{-}^{j,i-1}P_{tf,j,i-1,k}\right\|$$

where ${}^{j,i-1}P_{tn,j,i,k}=transl{(}^{j,i-1}T_{j,i}\cdot \left[{}^{j,i}P_{tn,j,i,k};1\right])$, and transl(⋅) denotes the operation that takes the first three rows of the fourth column of the matrix in parentheses.

#### 2.2.3. Differential Kinematics

The Jacobian matrix can relate the joint velocities of the CR to the corresponding velocities of the points on it, which is important for motion planning. The linear velocities and angular velocities of any point on the CR can be expressed as

(7) $${\dot{P}}_{any}=\left[\begin{array}{l} v_{any}\\ \omega_{any} \end{array}\right]=J_{{}_{any}}\cdot {\dot{Q}}_{any}$$

where ${\dot{P}}_{any}\in R^{6\times 1}$ represents the velocity of a random point on the CR, including the linear velocity, $v_{any}\in R^{3\times 1}$, and angular velocity, $\omega_{any}\in R^{3\times 1}$, and ${\dot{Q}}_{any}$ represents the velocity of the joint angles that have an effect on ${\dot{P}}_{any}$. Supposing that there are N Hooke joints with an effect on ${\dot{P}}_{any}$, we can obtain that

(8) $$\left[\begin{array}{l} v_{any}\\ \omega_{any} \end{array}\right]=\left[\begin{array}{ccccc} J_{1x}^{\left(v\right)} & J_{1y}^{\left(v\right)} & \cdots & J_{Nx}^{\left(v\right)} & J_{Ny}^{\left(v\right)}\\ J_{1x}^{\left(\omega \right)} & J_{1y}^{\left(\omega \right)} & \cdots & J_{Nx}^{\left(\omega \right)} & J_{Ny}^{\left(\omega \right)} \end{array}\right]\cdot {\left[\begin{array}{cccc} \begin{array}{cc} {\dot{\theta }}_{1x} & {\dot{\theta }}_{1y} \end{array} & \cdots & {\dot{\theta }}_{Nx} & {\dot{\theta }}_{Ny} \end{array}\right]}^{T}$$

where $J_{n}^{\left(v\right)}$ represents the transmission ratio of the linear velocity, $v_{any}$, $J_{n}^{\left(\omega \right)}$ represents the transmission ratio of the angular velocity, $\omega_{any}$, $\theta_{nx}$ and $\theta_{ny}$ represent the joint angles of joint n(n=1,2,⋯,N), and $J_{nx}^{\left(v\right)},$ $J_{ny}^{\left(v\right)},$ $J_{nx}^{\left(\omega \right)},$ and $J_{ny}^{\left(\omega \right)}$ can be expressed as follows:

(9) $$J_{nx}^{\left(v\right)}=x_{n}\times (P_{any}-P_{n})$$

(10) $$J_{ny}^{\left(v\right)}=y_{n}\times (P_{any}-P_{n})$$

(11) $$J_{nx}^{\left(\omega \right)}=\left\{ \begin{array}{c} T2R(T_{n-1}\cdot Trans_{z}(h_{u}))\cdot x,(n\geq 2)\\ T2R(Trans_{z}(h_{v}))\cdot x,(n=1) \end{array}\right.$$

(12) $$J_{ny}^{\left(\omega \right)}=\left\{ \begin{array}{c} T2R(T_{n-1}\cdot Trans_{z}(h_{u})\cdot Rot_{x}(\theta_{nx}))\cdot y,(n\geq 2)\\ T2R(Trans_{z}(h_{v})\cdot Rot_{x}(\theta_{1x}))\cdot y,(n=1) \end{array}\right.$$

where $P_{n}$ represents the position of joint n, $x_{n}$ and $y_{n}$ are the rotation axes of joint n, $x_{n}=J_{nx}^{\left(\omega \right)}$, $y_{n}=J_{ny}^{\left(\omega \right)}$, and $x={\left[1,0,0\right]}^{T}$ and $y={\left[0,1,0\right]}^{T}$ are the rotation axes in the local frame. According to the structure of the CR, the distances between adjacent Hooke joints are equal, except for the distance between the base and the 1st joint, we can obtain that $h_{u}=h_{1}$ and $h_{v}=h_{0}$. T2R(⋅) denotes the operation that takes the orthonormal rotation matrix of the matrix in brackets.

Due to the equivalence of joint angles within one segment, Equation (8) can be rewritten as follows:

(13) $$\left[\begin{array}{l} v_{any}\\ \omega_{any} \end{array}\right]=\left[\begin{array}{ccc} {\hat{J}}_{1} & \cdots & \begin{array}{cc} {\hat{J}}_{M} & {\hat{J}}_{extra} \end{array} \end{array}\right]\cdot {\left[\begin{array}{ccc} {\hat{\dot{Q}}}_{1} & \cdots & \begin{array}{cc} {\hat{\dot{Q}}}_{M} & {\hat{\dot{Q}}}_{extra} \end{array} \end{array}\right]}^{T}(M=\left[\frac{N}{3}\right])$$

where ${\hat{J}}_{m}\left(1\leq m\leq M\right)$, ${\hat{\dot{Q}}}_{m\left(1\leq m\leq M\right)}$, ${\hat{J}}_{extra},$ and ${\hat{\dot{Q}}}_{extra}$ are expressed as follows:

(14) $${\hat{J}}_{m}=\left[\begin{array}{l} \begin{array}{cc} \sum_{i=3(m-1)+1}^{3m}J_{ix}^{\left(v\right)} & \sum_{i=3(m-1)+1}^{3m}J_{iy}^{\left(v\right)} \end{array}\\ \begin{array}{cc} \sum_{i=3(m-1)+1}^{3m}J_{ix}^{\left(\omega \right)} & \sum_{i=3(m-1)+1}^{3m}J_{iy}^{\left(\omega \right)} \end{array} \end{array}\right](1\leq m\leq M)$$

(15) $${\hat{\dot{Q}}}_{m}=\left[\begin{array}{cc} \sum_{i=3(m-1)+1}^{3m}{\dot{\theta }}_{ix} & \sum_{i=3(m-1)+1}^{3m}{\dot{\theta }}_{iy} \end{array}\right](1\leq m\leq M)$$

(16) $${\hat{J}}_{extra}=\left\{ \begin{array}{c} \left[\begin{array}{c} \begin{array}{ccc} J_{(3M+1)x}^{\left(v\right)} & J_{(3M+1)y}^{\left(v\right)} & \begin{array}{cc} \cdots & \begin{array}{cc} J_{Nx}^{\left(v\right)} & J_{Ny}^{\left(v\right)} \end{array} \end{array} \end{array}\\ \begin{array}{ccc} J_{(3M+1)x}^{\left(\omega \right)} & J_{(3M+1)y}^{\left(\omega \right)} & \begin{array}{cc} \cdots & \begin{array}{cc} J_{Nx}^{\left(\omega \right)} & J_{Ny}^{\left(\omega \right)} \end{array} \end{array} \end{array} \end{array}\right],\mathrm{mod}(N,3)\neq 0\\ \;\;\;\;\;\;\;\;\;\;\;\;\;0,\mathrm{mod}(N,3)=0 \end{array}\right.$$

(17) $${\hat{\dot{Q}}}_{extra}=\left\{ \begin{array}{l} {}[{\dot{\theta }}_{(3M+1)x}\;{\dot{\theta }}_{(3M+1)y}\;\cdots \;{\dot{\theta }}_{Nx}\;{\dot{\theta }}_{Ny}],\mathrm{mod}(N,3)\neq 0\\ \;\;\;\;\;\;\;\;\;\;\;\;\;\;\;0,\mathrm{mod}(N,3)=0 \end{array}\right.$$

where mod(a,b) denotes the operation that takes the remainder of a divided by b. Hence, the Jacobian matrix, $J_{any}$, and the velocity of the joint angles, ${\dot{Q}}_{any}$, in Equation (7) can be found:

(18) $$J_{any}=\left[\begin{array}{ccc} {\hat{J}}_{1} & \cdots & \begin{array}{cc} {\hat{J}}_{M} & {\hat{J}}_{extra} \end{array} \end{array}\right]\in R^{6\times (2M+2\mathrm{mod}(N,3))}$$

(19) $${\dot{Q}}_{any}={\left[\begin{array}{ccc} {\hat{\dot{Q}}}_{1} & \cdots & \begin{array}{cc} {\hat{\dot{Q}}}_{M} & {\hat{\dot{Q}}}_{extra} \end{array} \end{array}\right]}^{T}\in R^{(2M+2\mathrm{mod}(N,3))\times 1}$$

## 3. The Pre-Grasping Motion Planning Method for the CR

In this section, we first describe the pre-grasping motion planning process for the CR and related assumptions. Next, we define the grasping workspace, which serves as the determining factor for initiating the pre-grasping phase. Then, we set the grasping area as the completion criteria for the pre-grasping phase. Finally we introduce the pre-grasping motion planning method based on an IAPF for the CR.

### 3.1. The Pre-Grasping Motion Planning Process for the CR

Non-cooperative target capture missions require a high level of reliability, particularly considering the mechanical effects that arise in a microgravity space environment, which can result in significant changes even under a small force. Therefore, it is imperative to incorporate a pre-grasping phase before there is contact between the CR and the target. In this phase, the CR, equipped with a variety of sensors, such as position and velocity sensors and vision sensors, moves to form a region known as the grasping area that restricts the movement of the target. Throughout the pre-grasping phase, the CR uses its position and velocity sensors to track the movement and speed of its joints and vision sensors to identify the location, shape, and size of the target. The target is treated as an obstacle during this process. The objective of the pre-grasping motion planning, facilitated by the sensor data, is to ensure that the body of the CR envelopes the target without causing a collision, forming a grasping area without a predetermined grasping configuration. Thus, the pre-grasping motion planning for the CR should guide the CR to move around the target and constantly verify if the grasping area has been successfully formed with the utilization of sensors. In this paper, we propose a pre-grasping motion planning method to guide the CR’s motion by constructing suitable potential fields.

The assumptions made in this paper are as follows:

- The CR is mounted on a spacecraft and can obtain geometric and motion information about the grasping target through its onboard sensors;
- The shape and size of the grasping target, detected by the vision sensors, fall within the grasping capabilities of the CR;
- The grasping target and the spacecraft are in a relative hovering state, determined by the Inertial Measurement Unit (IMU).

**Figure 5:**
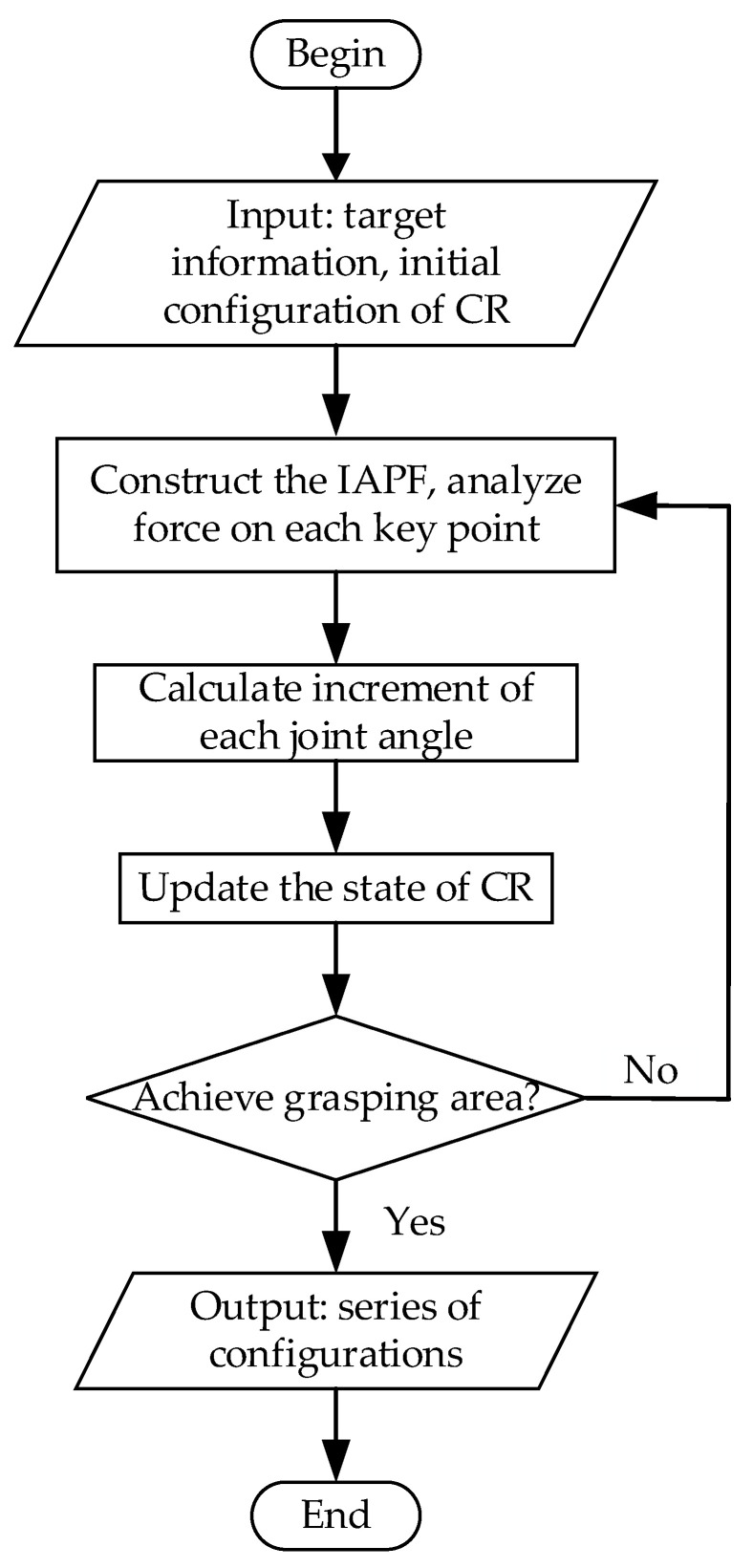
The flowchart of the pre-grasping motion planning process for the CR.

Based on these assumptions, the pre-grasping phase is initiated once the target is detected that has fallen within the grasping workspace. The pre-grasping motion planning process for the CR involves the following steps. Firstly, potential fields are constructed using the position of the target and the initial configuration of the CR. The increment of joint angles is then computed. Subsequently, the configuration is updated, and the formation of the grasping area is assessed. If the grasping area is successfully formed, the pre-grasping phase is concluded. Otherwise, the construction of potential fields continues, and the CR continues to move. A flowchart depicting the pre-grasping motion planning process for the CR is presented in Figure 5.

### 3.2. Grasping Workspace

To initiate the pre-grasping motion planning, it is crucial for the target to be in close proximity to the CR. In this paper, we define the term “grasping workspace” as the collective range of points where the target can be positioned when the CR is employed for pre-grasping tasks. The grasping workspace is determined by the joint limits and the size of the target. It is worth noting that the grasping workspace may differ for targets with varying shapes and sizes.

**Figure 6:**
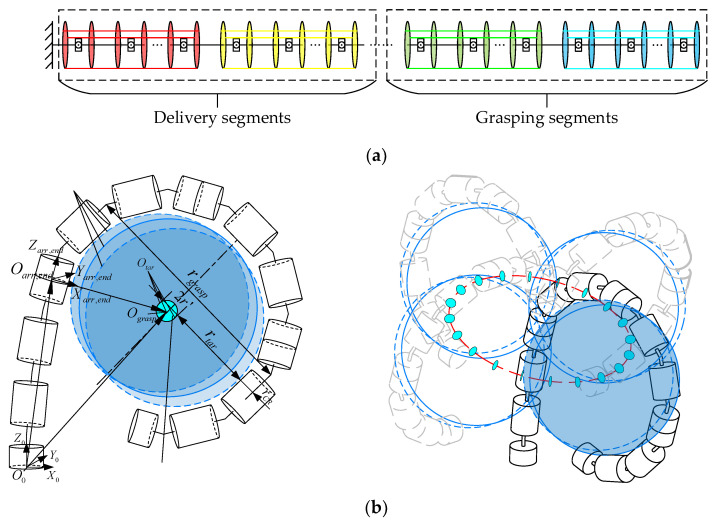
Schematic diagram of grasping workspace. (**a**) CR segment division illustration; (**b**) grasping workspace.

As shown in Figure 6a, according to different tasks, we consider that the segments of the CR are divided into two groups: grasping segments and delivery segments. The grasping segments are situated near the end, while the remaining segments are delivery segments. The primary function of the grasping segments is to create a grasping area and envelop the target by whole-arm grasping, while the delivery segments are primarily used to transport the grasping segments to be near the target. Therefore, the grasping segments can be considered as a gripper. Referring to our previous definition, the grasping workspace is the set of target positions that can be grasped by the CR; the grasping workspace in-plane is illustrated in Figure 6b (left). The blue circles in Figure 6b (left) represent the possible positions of the targets, and the position of the targets is denoted by $O_{tar}$. So, the set of $O_{tar}$ represents the grasping workspace. In Figure 6b (left), the cyan region centered at $O_{grasp}$ with a radius of $r^{\prime }$ is the set of $O_{tar}$, which illustrates the planar grasping workspace under the situation that one segment of the CR is used as the delivery segment. As for the spatial grasping workspace, as illustrated in Figure 6b (right), it is formed by rotating the blue circle around the axis $Z_{arr,end}$ for one complete revolution.

According to the analysis above, the grasping workspace is represented by the center and the boundary of the region as follows:

$O_{grasp}$ in the frame {arr,end} can be obtained as:

(20) $$\left\{ \begin{array}{l} {}^{arr,end}O_{grasp}={\left[r_{grasp}cos(\beta ),r_{grasp}sin(\beta ),0\right]}^{T}\\ {}^{arr,end}s={\left[r^{\prime }\cos(\gamma ),0,r^{\prime }\sin(\gamma )\right]}^{T} \end{array}\right.$$

where ${}^{arr,end}O_{grasp}$ is $O_{grasp}$ in the frame {arr,end}, $r_{grasp}$ is the radius of the inscribed circle of the region in which grasping segments formed, β is the angle rotating around the axis $Z_{arr,end}$ and β=[0,2π], ${}^{arr,end}s$ is the boundary of the grasping workspace in-plane, $r^{\prime }$ is the radius of the grasping workspace in-plane and will be calculated in Section 3.3, and γ is used to denote the grasping workspace in-plane, where γ=[0,2π].

Then the spatial grasping workspace is obtained as follows:

(21) $$C_{grasp}=transl(T_{arr,end}\cdot {[}^{arr,end}C_{grasp};1])$$

where $C_{grasp}$ is the spatial grasping workspace presented in the world coordinate system, ${}^{arr,end}C_{grasp}$ is the grasping workspace in the frame {arr,end}, where the coordinate system {arr,end} is located at the end of the delivery segments, and $T_{arr,end}$ is the homogeneous transformation matrix from the world coordinate system to the frame {arr,end}. Moreover, ${}^{arr,end}C_{grasp}$ can be expressed as:

(22) $${}^{arr,end}C_{grasp}=Rot_{z}(\beta )\cdot s{+}^{arr,end}O_{grasp}$$

After determining the number of delivery segments, $n_{arr}$, the set of $T_{arr,end}$ can be obtained using the forward kinematics and Monte Carlo method. In the following, we describe the process of obtaining $n_{arr}$. The total number of CR segments, denoted as $N_{CR}$, can be expressed as $N_{CR}=n_{arr}+n_{grasp}$. Here, the remaining segments after fulfilling the grasping requirements are utilized as delivery segments. Thus, it is essential to determine the number of grasping segments, $n_{grasp}$, based on information about the target.

To restrict the movement of the target, the grasping segments need to envelop the target so that the enveloping angle should exceed 180° without any collisions, which we call the grasping area. Further details regarding this analysis can be found in Section 3.3. Consequently, the calculation of $n_{grasp}$ can be derived as follows:

(23) $$n_{grasp}=\left\lceil \frac{(r_{tar}+r_{CR})\pi }{l_{CR}}\right\rceil +1$$

where $l_{CR}$ is the arc length corresponding to the inner tangent circle of one segment of the CR and $l_{CR}\in \left[0.3537,0.3900\right]$, ⌈⋅⌉ denotes upward rounding, $r_{tar}$ denotes the radius of the target, and $r_{CR}$ denotes the radius of the rigid links. In order to satisfy the demands of no collisions in the pre-grasping phase, one more segment is needed.

### 3.3. Grasping Area

#### 3.3.1. Grasping Area

Inspired by caging-based grasping [61], this paper aims to enable the CR to transition from the initial state to the enveloping state during the pre-grasping phase, constraining the target within an inescapable region and allowing subsequent grasping operations to be executed within a restricted area. Here, the term “grasping area” is defined as the region formed by the grasping segments that effectively limit the movement of the target. To reliably constrain the target and properly prepare for subsequent grasping operations, the plane in which the grasping area is contained is chosen to be perpendicular to the axis and passes through the centroid of the target.

**Figure 7:**
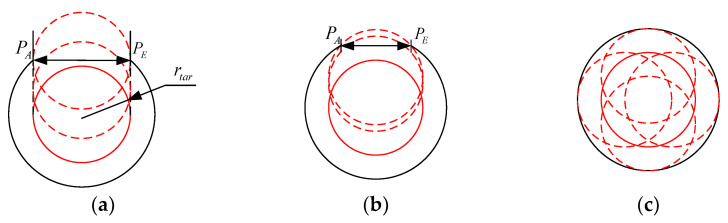
The relationships between the target and the CR. (**a**) $\|P_{A}P_{E}\|=2r_{tar}$; (**b**) $\|P_{A}P_{E}\|<2r_{tar}$; (**c**) $P_{A}\;\mathrm{and}\;P_{E}$ coincide.

Certain conditions must be met by the relationship between the grasping segments and the target to establish a grasping area. Figure 7 illustrates the three kinds of relationships between the grasping segments and the target. Assuming that at the time the grasping area has been formed and the pre-grasping phase has concluded, the next second the CR will curl up to grasp the target and make initial contact with the target. In Figure 7, the grasping segments are represented by a solid black line, the target by a red circle with a radius of $r_{tar}$, and the red dashed line indicates the potential positions where the target may be. $P_{A}$ denotes the position at the start of the grasping segments, which also serves as the end of the delivery segments, while $P_{E}$ represents the end of the CR.

1. When $\|P_{A}P_{E}\|=2r_{tar}$, the target has the potential to escape from the grasping area;
2. When $\|P_{A}P_{E}\|<2r_{tar}$, the target is effectively intercepted and unable to escape;
3. When $P_{A}$ and $P_{E}$ coincide, a closed region is formed, and the target is enclosed by the grasping segments, preventing it from escaping.

It is imperative to continually verify if the grasping area has been successfully formed during the pre-grasping motion planning process, and to determine the formation of the grasping area, $\|P_{A}P_{E}\|<2r_{tar}$ must be satisfied.

#### 3.3.2. Calculation of $r_{grasp}$

**Figure 8:**
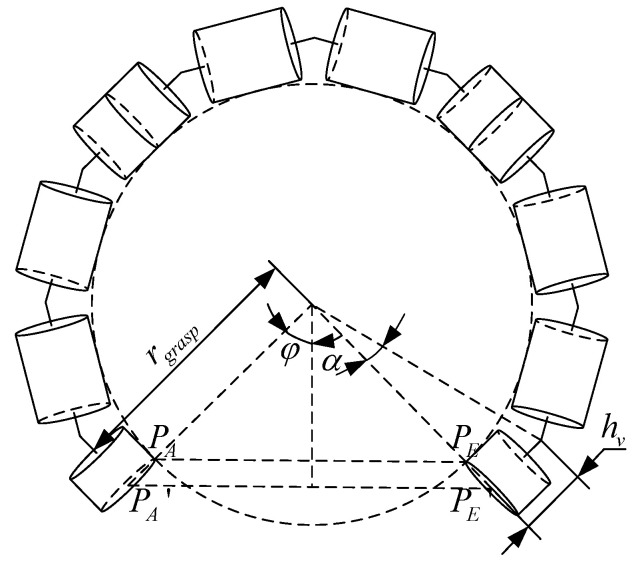
Schematic diagram of *r_grasp_*.

By defining the grasping area, we can calculate the radius, denoted as $r_{grasp}$, which corresponds to the arc formed by bending the backbone of the grasping segments for different targets. The target is in contact with the inner side of the grasping segments, so $P_{A}$ and $P_{E}$, as illustrated in Figure 8, refer to the inner side of the start and the end of the grasping segments, respectively.

Combining the analysis of the grasping area with Figure 8, we can obtain that

(24) $$||P_{A}P_{E}||=2(r_{grasp}-r_{CR})\sin(\varphi )<2r_{tar}$$

where φ is the central angle corresponding to the string $\left\|P_{A}P_{E}\right\|$ and α is determined by $tan\left(\alpha \right)=h_{v}/r_{grasp}$. According to the mechanism design of the CR, the relationship between α and φ can be expressed as

(25) $$2\varphi =2\pi -6n_{grasp}\alpha$$

Combining Equations (14) and (15), we can obtain that

(26) $$(\frac{h_{v}}{tan(\alpha )}-r_{CR})\sin(\varphi )<r_{tar}$$

By replacing α with φ in inequality (26), we can calculate the maximum value of φ that satisfies the given constraints. Substituting this value of φ into Equation (24) allows us to compute the maximum value of $r_{grasp}$.

### 3.4. Pre-Grasping Motion Planning Method Based on IAPF

#### 3.4.1. IAPF Construction

- Spatial rotating potential field:

The target is regarded as a central star and the end of the CR as a planet orbiting around the star. The end of the CR experiences a force that is perpendicular to the line connecting the center of the target and the end of the CR, which drives the end to lead the rest of the CR to encircle the target. The force generated by the rotating potential field is denoted as $F_{rot}$ and can be expressed as

(27) $$F_{rot}=n_{rot}\cdot f_{rot}$$

where $f_{rot}$ is the value of $F_{rot}$ and $n_{rot}$ is the direction of $F_{rot}$, and they are obtained as follows:

(28) $$f_{rot}=\frac{K_{rot}}{\left|p_{end,proj}-O_{tar}\right|}$$

(29) $$n_{rot}=\frac{p_{end,proj}-O_{tar}}{\left|p_{end,proj}-O_{tar}\right|}\times n_{tar}$$

where $K_{rot}$ represents the gain coefficient in a rotating potential field, $p_{end,proj}$ is the projection of the end of the CR on the plane that is perpendicular to the target axis and passing through the center of the target, and $n_{tar}$ is the central axis of the target, the direction of which is determined by the right-hand rule.

- Attractive potential field:

A traditional attractive potential field is used for driving the robot to reach the target position, which is generated by the positional relationship between a set target point and the robot. In this paper, since there is no specific set target point, instead, a rotating potential field is employed to propel the end of the CR. However, only carrying this out under the effect of the rotating potential field creates a hindrance to achieving a grasping area. To expedite the process of moving the end of the CR towards the target and swiftly establish a grasping area, a position-based attractive potential field with a finite range of influence is constructed by regarding the center of the target as a virtual target point. The range of the attractive field extends from infinity to a distance $\rho_{att}$ away from the center of the target. The generated attractive force is proportional to the distance between the virtual target point and the end of the CR, with the attractive force becoming zero when the end is on the boundary of the influential range. Therefore, the attractive force, $F_{attp},$ exerted on the end of the CR can be expressed as follows:

(30) $$F_{attp}=\left\{ \begin{array}{c} K_{attp}(O_{tar}-P_{end}-\rho_{att}),|O_{tar}-P_{end}|>\rho_{att}\\ \;\;\;\;\;\;\;\;0,|O_{tar}-P_{end}|\leq \rho_{att} \end{array}\right.$$

where $K_{attp}$ represents the gain coefficient in an attractive potential field.

The above position-based attractive potential field can guide the CR to form a grasping area. It works well in 2D plane motion planning, where the target and the robot are in-plane and the normal vector of the grasping area is parallel to the axis of the target. However, a planning result that does not consider the posture of the grasping area will result in a large angle between two axes and insufficient safety distance between the links of the CR and the target, which may lead to failure and reduce the operability of subsequent grasping operations. The ideal plane where the grasping area is located should be perpendicular to the axis of the target and pass through the centroid of the target. Therefore, we add a posture potential field to make the plane formed by the grasping segments close to the ideal plane. In other words, the posture potential field can make the normal vector of the plane formed by grasping segments approximately parallel to the axis of the target. It is known that the normal of a plane defined by two axes can be obtained by taking the cross product of two axes. Thus, the normal vector of the plane formed by the grasping segments is defined as follows:

(31) $$\xi_{g}=\left\{ \begin{array}{c} {\left[\begin{array}{c} \left(P_{(n_{CR}-n_{grasp}),end}-P_{(n_{CR}-n_{grasp}-1),end})\times (P_{(n_{CR}-n_{grasp}),end}-P_{(n_{CR}-n_{grasp}+1),end}\right)\\ \vdots \\ \left(P_{(n_{CR}-1),end}-P_{(n_{CR}-2),end})\times (P_{(n_{CR}-1),end}-P_{n_{CR},end}\right) \end{array}\right]}^{T},(n_{grasp}>1)\\ \;\;\;\;\;\;\;\;\;\;\;\;\;\;(P_{n_{CR},2}-P_{n_{CR},0})\times (P_{n_{CR},2}-P_{n_{CR},end}),(n_{grasp}=1) \end{array}\right.$$

The attractive force of the posture potential field can be expressed as

(32) $$F_{atto}=K_{atto}\sum \left({\bar{\xi }}_{g}-{\bar{\xi }}_{tar}\right)$$

where $K_{atto}$ represents the gain coefficient in the posture potential field, ${\bar{\xi }}_{g}\in R^{3\times 1}$ when $n_{grasp}=1$ or ${\bar{\xi }}_{g}\in R^{3\times \left(n_{grasp}-1\right)}$ represents the result of the normalization of each column in $\xi_{g}$, and ${\bar{\xi }}_{tar}\in R^{3\times 1}$ represents the normalization of the axis of the target, denoted as $\xi_{tar}$.

- Repulsive potential field:

Due to the fact that the target is deemed an obstacle, it is necessary to determine whether the segments of the CR are within a secure distance, which is a distance away from the center of the target, denoted as $\rho_{rep}$. The part of the CR within $\rho_{rep}$ will be subjected to the influence of the repulsive force generated by the repulsive potential field, causing it to relocate to outside of $\rho_{rep}$. Consequently, the repulsive force exerted on each key point of the CR can be expressed as follows:

(33) $$F_{rep,i}=\left\{ \begin{array}{c} K_{rep}{\left(\frac{1}{\left|P_{CR,i}-O_{tar}\right|}-\frac{1}{\rho_{rep}}\right)}^{2}\frac{P_{CR,i}-O_{tar}}{\left|P_{CR,i}-O_{tar}\right|},|P_{CR,i}-O_{tar}|\leq \rho_{rep}\\ \;\;\;\;\;\;\;\;\;\;\;0,|P_{CR,i}-O_{tar}|>\rho_{rep} \end{array}\right.$$

where $K_{rep}$ represents the gain coefficient in the repulsive potential field and $P_{CR,i}$ represents the position of ith key point of the CR.

**Figure 9:**
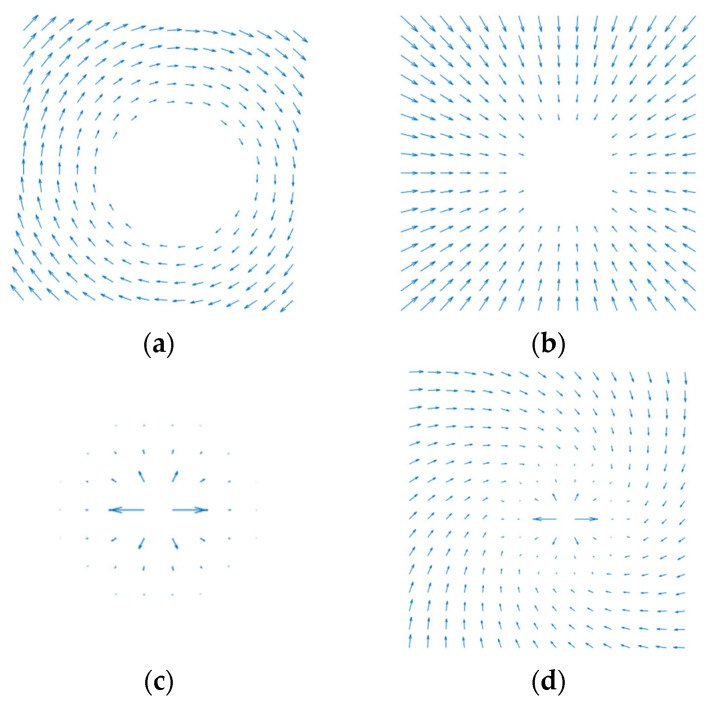
The distribution diagram of potential fields. (**a**) Rotating potential field; (**b**) attractive potential field; (**c**) repulsive potential field; (**d**) resultant potential field.

The distribution diagrams of the potential fields are illustrated in Figure 9. It can be observed that under the influence of the rotating potential field illustrated in Figure 9a, the end of the CR will move along the circumference, gradually surrounding the target. However, as depicted in Figure 9d, with the combined effect of all the potential fields mentioned above, the end of the CR can rapidly approach and enclose the target. In addition, the APF may lead the robot to be trapped in the local minimal position. But, with a rotating potential field added in this method, which is mainly used to drive the CR to encircle the target, the combined potential fields can always drive the CR to move around the target with the proper parameters of the potential fields.

#### 3.4.2. Relate Forces to Joint Angles

We apply the maximum work planning strategy to map the forces in the task space to the joint angles in the joint space. Doing the maximum work in each planning period makes the potential energy drop most rapidly, and the CR moves rapidly to achieve the grasping area. Specifically, firstly, the force generated by the resultant potential field on each key point, $P_{i}\left(i=1,2,\cdots ,m\right)$, of the CR should be analyzed and calculated. Then, the gradient of the total work can be calculated through $\nabla W=\sum F_{i}^{T}J_{i}$. By making $\dot{Q}$ be equal to the gradient of the total work, ∇W, the increment of the joint angles can be obtained as follows:

(34) $$\left[\begin{array}{cc} \Delta q_{1,x} & \begin{array}{cccc} \Delta q_{1,y} & \cdots & \Delta q_{n_{CR},x} & \Delta q_{n_{CR},y} \end{array} \end{array}\right]=(F_{rot}^{T}+F_{attp}^{T})J_{end}^{\left(v\right)}+F_{atto}{}^{T}J_{end}^{\left(\omega \right)}+\sum_{i=1}^{m}F_{rep,i}{}^{T}J_{i}^{\left(v\right)}$$

Thus, the new set of CR joint angles is expressed as

(35) $$Q_{new}=Q_{cur}+\delta t\cdot \Delta Q$$

where $Q_{cur}$ are the current CR joint angles, δt is the length of each step, and ΔQ is expressed in Equation (25).

## 4. Simulation

To verify the performance of the proposed method, this section presents simulations of the grasping workspace and motion planning process. Given a specific initial configuration, $C_{0},$ of the CR and information about the target, simulations of pre-grasping motion planning are carried out in planar and spatial scenarios under the following initial settings:

- The simulations are carried out on a four-segment CR. Each segment consists of three Hooke joints and four links. The length of the links is $h_{0}=h_{3}=0.065\;m$ and $h_{1}=h_{2}=0.13\;m$. The limit of the joint angle is $q_{max}=\frac{\pi }{3}$. The radius of the CR is $r_{CR}=0.035\;m.$
- The calculation for the repulsive force requires knowledge about the distance between the target and the key points of the CR. Notably, the sampling density of these points impacts both the computational efficiency and planning results. For the following simulations, 30 key points are evenly distributed along each segment of the CR, spaced at 13 mm intervals.
- The corresponding parameters of the potential field are set as $\rho_{att}=0.05\;m$, $\rho_{rep}=0.045\;m$, $K_{rot}=2$, $K_{attp}=K_{atto}=2$, and $K_{rep}=0.5$.
- Typically, the morphology of the CR is represented by its backbone. To simplify the calculation in the simulation, the target is expanded by adding $r_{CR}$ to its radius. In the results of the simulation are represented below; the actual size of the target is demonstrated in dark gray, and the expanded size appears in light gray. The red centerline represents the axis of the spatial target.

### 4.1. Grasping Workspace

**Figure 10:**
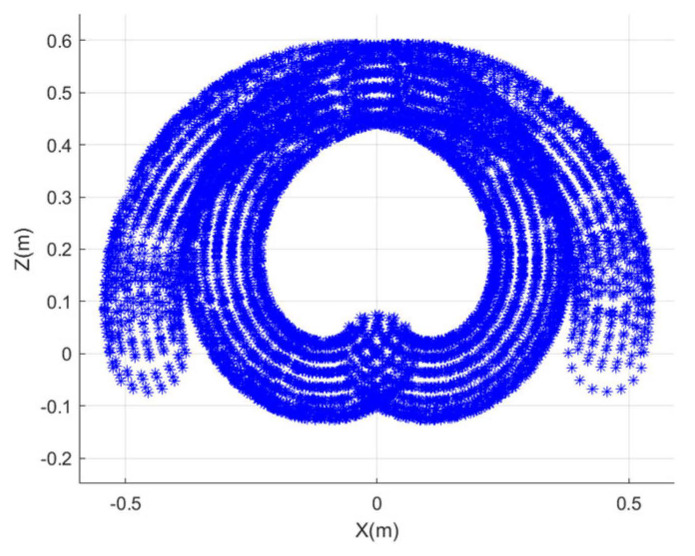
Planar grasping workspace in x−z plane.

**Figure 11:**
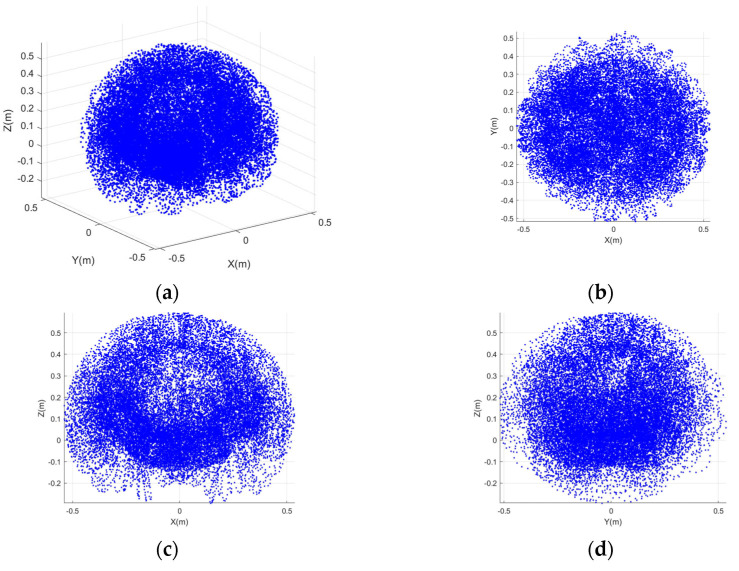
Spatial grasping workspace. (**a**) Overall view; (**b**) in *x* − *y* plane; (**c**) in *x* − *z* plane; (**d**) in *y* − *z* plane.

From $q_{max}=\frac{\pi }{3}$, the maximum bend angle for one segment in the plane is extrapolated as π. With the designed structure, the value of $r_{tar}$ can be obtained according to the discussion in Section 3.3, and $r_{tar}=\left[0.07759,0.33363\right]\;m$. Assuming that $r_{tar}=0.12\;m$, $n_{grasp}=3$ and $n_{arr}=1$ are obtained based on Section 3.2. Equation (26) can be simplified based on a third-order Taylor series expansion, then we can obtain that $r_{grasp}=0.2299\;m$, $r^{\prime }=0.0749\;m$. The workspace of the delivery segment is characterized utilizing the Monte Carlo method. Derivatively, the grasping workspace is obtained based on the method in Section 3.2, as shown in Figure 10 and Figure 11, which illustrate the planar and spatial grasping workspace for the CR respectively, with $r_{tar}=0.12\;m$.

### 4.2. Pre-Grasping Motion Planning

At the beginning of the pre-grasping phase, the target is situated within the grasping workspace. Therefore, the position of the target, $P_{tar},$ is selected based on the result computed in Section 4.1. Moreover, the initial configuration, $C_{0},$ of the CR must avoid singular configurations. According to Section 3.3, the criterion to determine if the grasping area has been successfully formed is $\|P_{A}P_{E}\|<2r_{tar}$. The following simulations are conducted for cases where the target is positioned within a plane and in three-dimensional space. In the first simulation, pre-grasping motion planning is executed for the target of varying $r_{tar}$ within a plane. In the second simulation, pre-grasping motion planning is conducted for the same target in space to investigate the impact of the posture potential field on the results of the pre-grasping motion planning.

#### 4.2.1. Motion Planning for Planar Targets

In the subsequent simulation, the pre-grasping motion planning is conducted for targets located within a plane, with $r_{tar1}=0.12\;m$ and $r_{tar2}=0.16\;m$. Consequently, $n_{grasp}=3$, $n_{arr}=1$, $r_{grasp1}=0.2299\;m$, $r_{grasp2}=0.2490\;m$, $r_{1}^{\prime }=0.0749\;m$, and $r_{2}^{\prime }=0.0540\;m$ are calculated based on $r_{tar1}$ and $r_{tar2}$. As for the initial configuration, $C_{0}$, we set it as [0,−0.2,0,0.15,0,0.15,0,0.2], and an identical $P_{tar}$ is employed for the planning and simulation.

**Figure 12:**
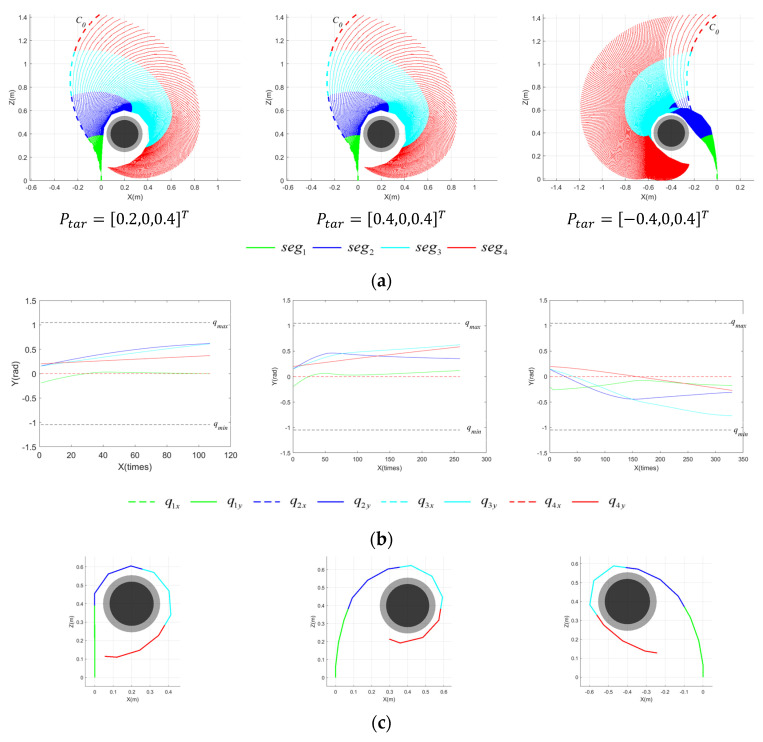
Motion process of CR for planar targets at varying $P_{tar}$ with $r_{tar1}=0.12\;m.$ (**a**) Motion process of CR; (**b**) variation in joint angles; (**c**) final configuration of CR.

**Figure 13:**
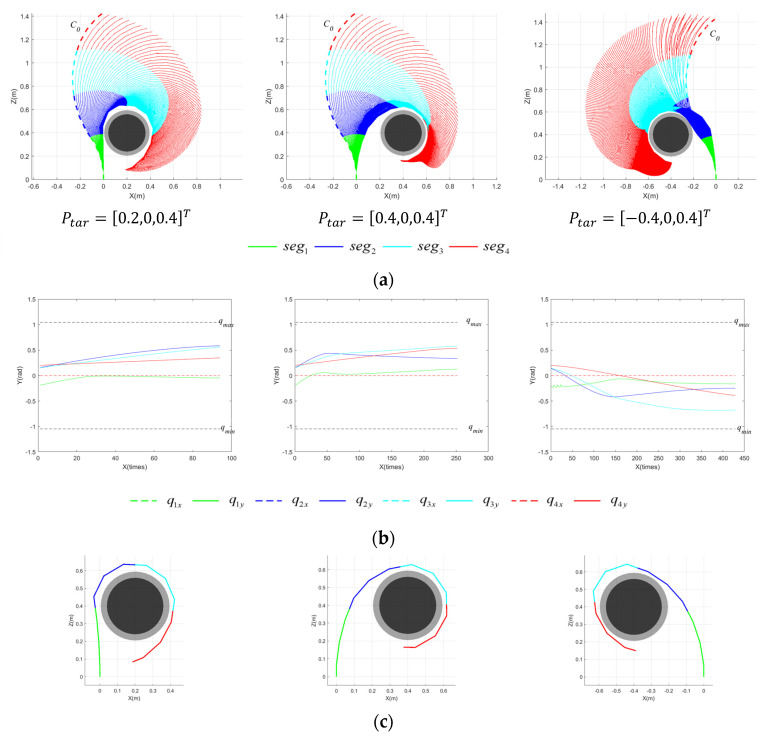
Motion process of CR for planar targets at varying $P_{tar}$ with $r_{tar2}=0.16\;m.$ (**a**) Motion process of CR; (**b**) variation in joint angles; (**c**) final configuration of CR.

Figure 12 shows the pre-grasping motion process of the CR for planar targets with $r_{tar1}$ located at three different positions within the grasping workspace. Figure 12a illustrates the process of the CR forming the grasping area, influenced by the IAPF. Each segment is distinguished by different colors, while the thick, dashed lines represent the initial configuration, $C_{0}$. Figure 12b displays the variation in joint angles of each segment throughout the motion process. The dashed lines reflect the change in $q_{j,x}$ with the number of execution times, while the solid lines represent the change in $q_{j,y}$. The upper and lower limits of the joint angle, denoted as $q_{max}$ and $q_{min}$, are signified by black dashed lines, and $q_{min}=-q_{max}$. Figure 12c presents the final configuration of the CR. Figure 13 captures the pre-grasping motion process of the CR for the target with $r_{tar2}$ under the same settings. This simulation validates that the proposed grasping workspace is instructive for pre-grasping motion planning. Given various positions and targets within a plane, the CR can successfully form a grasping area based on potential fields. The analysis of the result indicates that the execution times required are comparable in the first two positions, despite different radii, while the third position requires more execution times for $r_{tar2}$ than $r_{tar1}$. The complexity of the CR’s motion arises from the coupling of multiple joints, and the CR has multiple degrees of freedom. As a result, achieving optimal results for a feasible path and moving in the steepest descent direction may be challenging.

#### 4.2.2. Motion Planning for Spatial Targets

As mentioned in the preceding section, pre-grasping motion planning is executed for planar targets. The plane where the grasping area is contained is perpendicular to the axis of the target, and it passes through the centroid of the target. As such, the subsequent simulations are conducted on spatial targets constrained by a fixed radius of $r_{tar}=0.12\;m$, with an identical initial configuration, $C_{0}=\left[0.05,0.05,0.05,0.15,-0.05,0.15,0,0.2\right]$, but they differ in $P_{tar}$.

**Figure 14:**
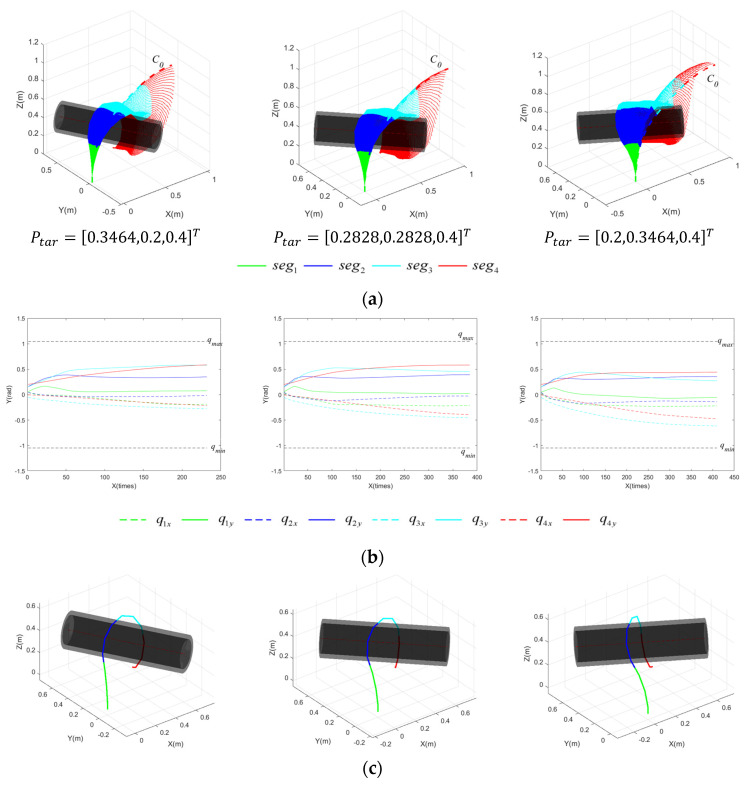
Motion process of CR for spatial targets at varying $P_{tar}$ with $r_{tar}=0.12\;m$, disregarding posture potential field. (**a**) Motion process of CR; (**b**) variation in the joint angles; (**c**) final configuration of CR.

**Figure 15:**
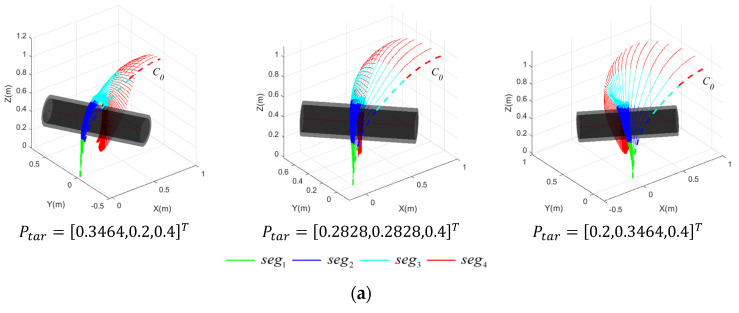

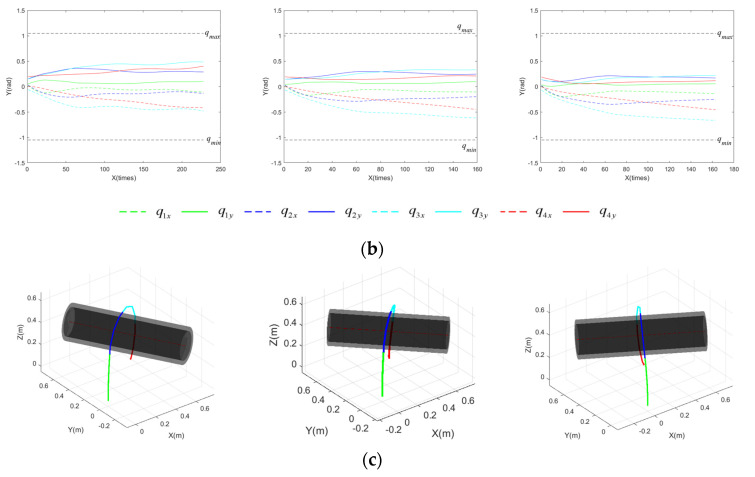
Motion process of CR for spatial targets at varying $P_{tar}$ with $r_{tar}=0.12\;m,$, considering a posture potential field. (**a**) Motion process of CR; (**b**) variation in the joint angles; (**c**) final configuration of CR.

Figure 14 presents the motion planning results of the CR for cylindrical targets devoid of a posture potential field, including the motion process of the CR forming the grasping area, the variation in joint angles of each segment throughout the motion process, and the final configuration of the CR when the grasping area is achieved. Conversely, Figure 15 illustrates the pre-grasping motion planning results of the CR under the influence of a posture potential field.

As shown in Figure 14 and Figure 15, successful pre-grasping motion planning is achieved within a limited iteration count. However, it is obvious that the execution times are greatly reduced with a posture potential field taken into account in the last two simulations, and the joint angles vary in a smaller range. It is noticeable that the normal vector of the plane formed by the grasping segments of the CR, as depicted in Figure 14c, deviates substantially from the axis of the target. The discrepancy between the two is considerably reduced in Figure 15. To better explain how the posture potential field affects the results of pre-grasping motion planning for the CR, we have developed a new metric—the average axis error, $\xi_{err,ave}$. This facilitates comprehending the difference between the plane constituted by the grasping segments and the ideal plane in which the grasping area should be contained. $\xi_{err,ave}$ is defined as follows:

(36) $$\xi_{err,ave}=norm(\frac{\sum \left({\bar{\xi }}_{g}-{\bar{\xi }}_{tar}\right)}{n_{grasp}-1})$$

where norm(⋅) denotes the calculation of the Euclidean norm for the vector in brackets.

Table 2 presents a comparison of $\xi_{err,ave}$ when the posture potential field is included and when it is not. It can be observed that the $\xi_{err,ave}$ is reduced under the influence of the posture potential field, indicating that the posture potential field is effective in directing the grasping segments of the CR to move toward an ideal plane. And the difference, $\xi_{err,ave},$ between the two methods is more pronounced in P2 and P3, which is due to the fact that the execution stops when the grasping area is achieved. The more difficult it is to achieve a grasping area without a posture potential field, the more obvious the difference between the two methods is. This indicates that a posture potential field is more effective when pre-grasping motion planning is implemented on spatial targets.

To more comprehensively illustrate the influence of the posture potential field on the pre-grasping motion planning of the CR, a comparison is carried out between the two algorithms. Specifically, 1000 rounds of pre-grasping motion planning are carried out using two different algorithms: one that includes a posture potential field and another that does not. The parameters of the potential fields and $C_{0}$ are identical for each run, except for the potential fields included in different algorithms. At the beginning of each round, a random spatial pose of the target with $r_{tar}=0.12\;m$ is chosen within the grasping workspace. To this end, the position of the target, referred to as $P_{tar}$, is designated as $\left\{P_{tar}|0.2\leq P_{tar,x}\leq 0.4,-0.2\leq P_{tar,y}\leq 0.2,0.3\leq P_{tar,z}\leq 0.4\right\}$. The rotation angle of the target around the *z*-axis is limited to $\left[\frac{\pi }{6},\frac{\pi }{3}\right]$. Then, pre-grasping motion planning is conducted using two algorithms, respectively. The results of the different algorithms are recorded, including $\xi_{s}$ to indicate whether the motion planning is successful, $\xi_{err,ave}$, and $\xi_{n},$ which represents the execution times. It is noticeable that the number of maximum execution times is set to 500 for each run. $\xi_{s}$ is set to 1 if the grasping area is achieved within the limited execution times, otherwise $\xi_{s}$ is set to 0. At the end, a comparison is carried out with the success rate, $\xi_{sr}$; the average of $\xi_{err,ave},$ presented as ${\bar{\xi }}_{err,ave}$; and the average execution times, which are presented as ${\bar{\xi }}_{n}$. $\xi_{sr}$, ${\bar{\xi }}_{err,ave}$ and ${\bar{\xi }}_{n}$ and are obtained as follows:

(37) $$\xi_{sr}=\frac{\sum_{i=1}^{n_{g}}\xi_{s,i}}{n_{g}}$$

(38) $${\bar{\xi }}_{err,ave}=\frac{\sum_{i=1}^{n_{g}}\xi_{s,i}\xi_{err,ave,i}}{\sum_{i=1}^{n_{g}}\xi_{s,i}}$$

(39) $${\bar{\xi }}_{n}=\frac{\sum_{i=1}^{n_{g}}\xi_{s,i}\xi_{n,i}}{\sum_{i=1}^{n_{g}}\xi_{s,i}}$$

where $n_{g}$ is the number of groups in which motion planning algorithms are conducted, $n_{g}=1000$, and $\xi_{X,i}$ is the $\xi_{X}$ of the ith record.

The results are listed in Table 3. It is noticeable that ${\bar{\xi }}_{err,ave}$ and ${\bar{\xi }}_{n}$ are computed for successful planning instances.

As observed in Table 3, the success rate of pre-grasping motion planning for the CR is superior when under the influence of the posture potential field. Additionally, the value of $\xi_{err,ave}$ is lower, and the average number of calculations is reduced, indicating greater efficiency. The posture potential field effectively drives the CR to move to encircle the spatial targets. A smaller $\xi_{err,ave}$ indicates that the body of the CR is further away from the target, and the CR has more space to move in the next step and a higher probability of success. Thus, when the target is positioned spatially, the pre-grasping motion planning method employing the posture potential field is safer and more effective.

However, a success rate of 100% is not achieved with either method, despite all $P_{tar}$ selections being within the grasping workspace. This discrepancy arises due to the grasping workspace and the parameters of the potential fields. The calculation for the grasping workspace is still challenging for whole-arm grasping because the CR moves flexibly and each part of the CR can be a manipulator. So far, there is no unified method for calculating the grasping workspace for the CR. In this paper, a simplified grasping workspace is calculated solely by factoring in the center of the target, disregarding the effects of variations in the target’s posture. Consequently, the motion planning may fail when $P_{tar}$ is situated on the boundary of the grasping workspace. Moreover, the calculations were implemented with fixed potential field coefficients and a fixed step size. Although small parameters could lead to success, it would cost too many execution times, and it would fail under a limitation of execution times. An adaptive adjustment of these parameters could optimize the motion planning results.

## 5. Conclusions

In this paper, we present a pre-grasping motion planning method for the CR based on an IAPF considering the impact of microgravity environments in space. The IAPF is utilized to guide the CR to encircle the target without predetermined goal configurations during the pre-grasping phase. By using a spatial rotating potential field, the CR moves to encircle the target. The use of the attractive potential field enhances the performance of the pre-grasping motion planning. Simulations are conducted for planar and spatial targets, verifying the feasibility and effectiveness of the proposed method. The implementation of a posture potential field for the spatial targets increased the success rate of the pre-grasping motion planning to 97.8%. The method proposed in this paper can be applied to other types of CRs for whole-arm grasping tasks that require safety and reliability. This work can lay a foundation for CRs to be utilized for subsequent grasping operations on ADR missions in the future.

However, this paper does not consider the adaptive adjustment of the parameters of potential fields and optimization. Moreover, the fact that the CR has a floating base in space is not taken into account in this paper. In the future, we will consider the force applied to driving cables and the influence of floating bases to optimize the results of pre-grasping motion planning for further exploration of space applications.

## Figures and Tables

**Table 1:** Parameters of the CR.

Parameter Names	Parameter Values
Enveloped size (m)	Φ0.07×1.56
Segments	4
Hooke joints of each segment	3
DOFs of CR	8
Driving cables of CR	12

**Table 2:** $\xi_{err,ave}$ of the cases with and without a posture potential field.

Methods	P1 *	P2 *	P3 *
With a posture potential field	0.46488	0.44368	0.40888
Without a posture potential field	0.14494	0.06222	0.02380

* P1 represents $P_{tar}={\left[0.3464,0.2,0.4\right]}^{T}$; P2 represents $P_{tar}={\left[0.2828,0.2828,0.4\right]}^{T}$; P3 represents $P_{tar}={\left[0.2,0.3464,0.4\right]}^{T}$.

**Table 3:** Comparison of the cases with and without a posture potential field.

Methods	$\xi_{sr}$	${\bar{\xi }}_{err,ave}$	${\bar{\xi }}_{n}$
With a posture potential field	72.6%	0.51197	208.75
Without a posture potential field	97.8%	0.16029	113.16

## Data Availability

Data are contained within the article.
